# High-Pressure Injection Injuries to the Face: An Unusual Case, Review of the Literature, and Proposed Management Algorithm

**DOI:** 10.7759/cureus.86786

**Published:** 2025-06-26

**Authors:** Henry Miller, Martin Buta, Eric Emberton, Kavitha Ranganathan, Doug Dembinski

**Affiliations:** 1 Plastic and Reconstructive Surgery, Mass General Brigham, Harvard Medical School, Boston, USA; 2 Plastic and Reconstructive Surgery, Massachusetts General Hospital, Boston, USA; 3 Plastic and Reconstructive Surgery, Brigham and Women's Hospital, Harvard Medical School, Boston, USA

**Keywords:** craniofacial trauma, high-pressure injection injury, oculoplastic surgeries, penetrating facial trauma, plastic and reconstructive surgery

## Abstract

High-pressure injection injuries to the hand are well-described, but similar injuries to the face are far less common and sporadically reported. Due to the complexity of facial anatomy and benign initial clinical presentation, these injuries can be underappreciated, leading to a delay in treatment and undesirable outcomes. In this paper, the authors present an unusual case of a pressurized diesel fuel injection injury to the face via an entry wound at the base of the nare. The caustic fuel caused widespread liquefactive necrosis of the subcutaneous fat of the face, orbit, and neck, requiring serial operative debridements by plastic surgery, oculoplastic surgery, and otolaryngology, respectively. Following a 20-day hospital course and seven total operations, the patient was discharged home with an infraorbital wound resulting in ectropion, requiring ectropion release and repair and full-thickness skin grafting. When managing injection injuries to the face, it is important to determine the exact mechanism of injury and appreciate the severity of underlying soft tissue damage that can progress quickly with delayed intervention. In most cases, early operative intervention is required, as well as early multidisciplinary care involving intensivists and ophthalmology, to optimize outcomes.

## Introduction

Pressurized injection injuries to the face are a rare clinical entity that can be highly morbid and challenging to manage. Cases have been reported in the literature beginning in 1964 with a grease gun injury to the eye [1]. Since that time, 32 cases of facial injection injuries have been reported, with injected substances ranging from air and water to the more caustic paint, diesel fuel, and grease. As with pressurized hand injuries, which have been written about extensively, facial injection injuries frequently affect male manual laborers [2]. These injuries constitute a surgical emergency, requiring prompt recognition and early intervention to preserve vision and prevent extensive soft tissue loss and necrosis. We present a case of a facial injection injury with entry site at the alar base, ultimately requiring a complex hospital course with multiple operations. We performed a literature review of all facial injection injuries and have proposed a workup and treatment algorithm.

## Case presentation

**Figure 1:**
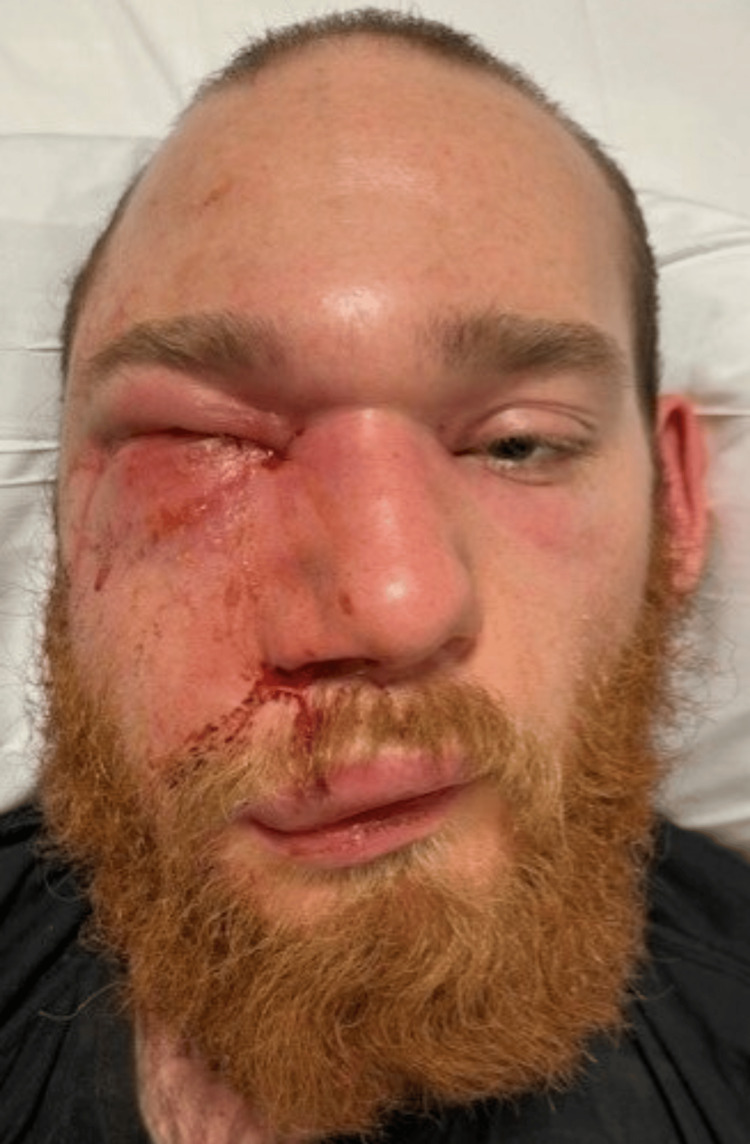
Photo of the patient upon presentation to the emergency department post injury day two

An otherwise healthy 25-year-old male, heavy equipment mechanic, presented to an outside hospital after sustaining an injection injury to the base of the right nare while checking a cylinder containing diesel fuel pressurized at approximately 500 PSI. He underwent an urgent maxillofacial CT scan demonstrating diffuse subcutaneous swelling and emphysema extending from the mandible to the calvarium with involvement of the inferior orbit. He subsequently received steroids and antibiotics and underwent a right lateral canthotomy with a local oculoplastic surgeon due to concern for orbital compartment syndrome. He was then transferred to our institution for escalation of care. He was admitted to the intensive care unit and was evaluated by the plastic surgery and ophthalmology teams. On exam, he had significant right facial soft tissue edema, including the upper and lower eyelids, with mild facial erythema, and right facial nerve palsy (Figure 1). The patient was initially managed conservatively with serial exams.

**Figure 2:**
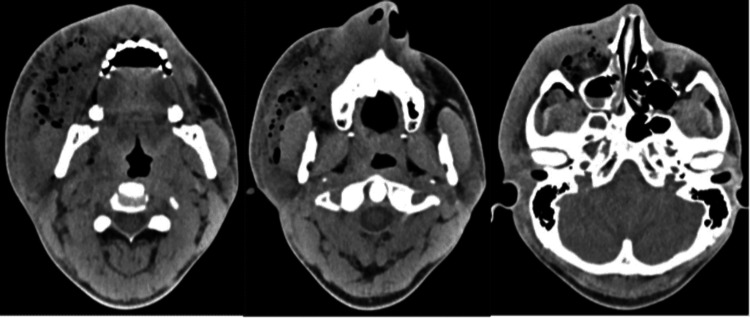
Facial CT on post injury day two with extensive subcutaneous emphysema on R face extending from the mandible to the calvarium with involvement of the inferior orbit

**Figure 3:**
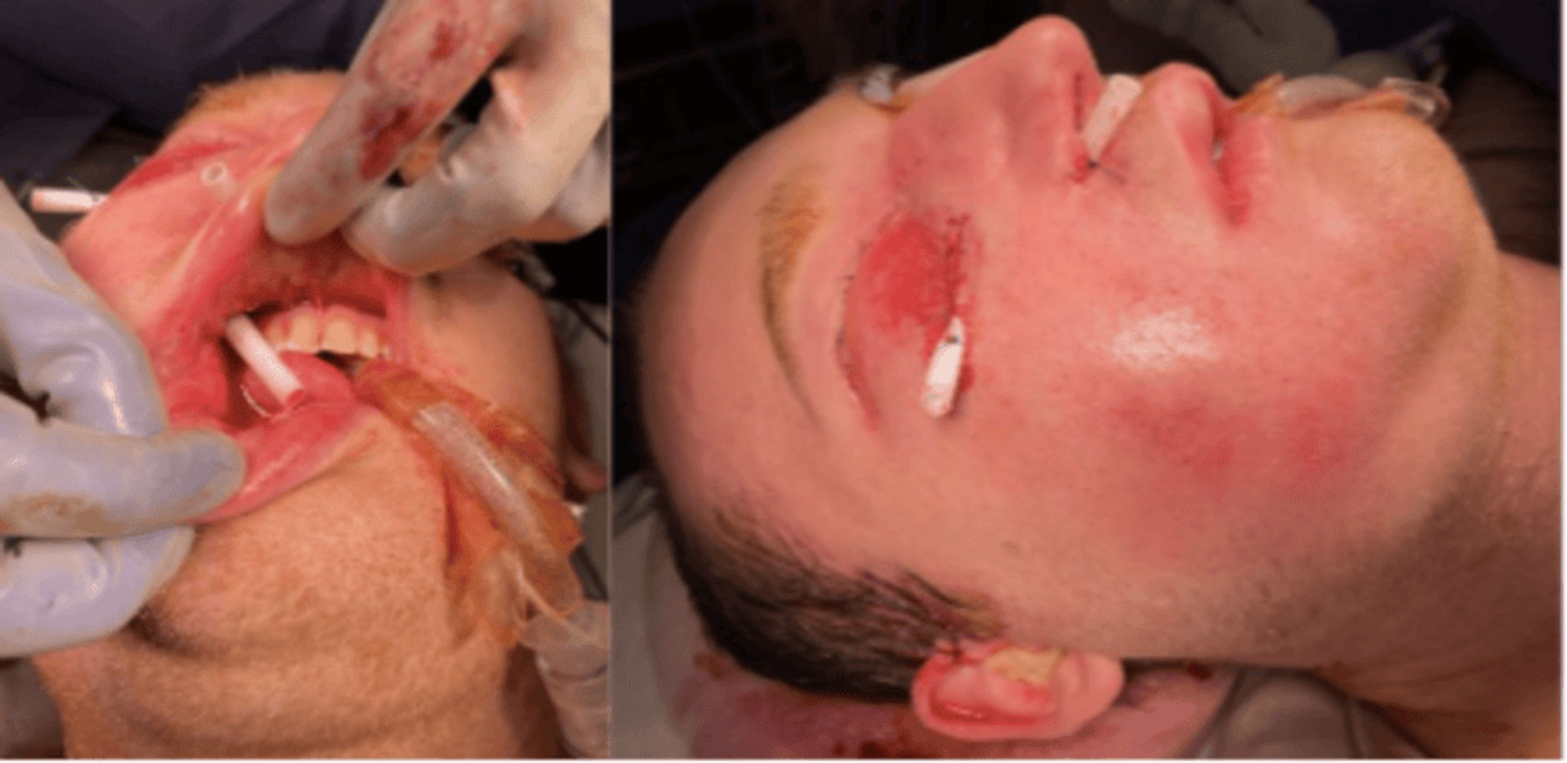
Intra-operative photo at index operation following washout and drain placement

On post injury day three, repeat maxillofacial CT demonstrated persistent right face diffuse swelling and subcutaneous gas extending into the right orbit (Figure 2). He developed a progression of facial swelling with severe and unremitting pain and reported a strong odor of diesel fuel with the sensation of trickling down his throat. Given these findings, he underwent emergent operative exploration with plastic surgery. An initial incision was made in the right upper buccal sulcus, with the immediate evacuation of turbid fluid, which was sent for culture. Exploration of the wound demonstrated significant liquefactive necrosis of the subcutaneous tissues of the right face with preservation of the superficial skin. An additional counter-incision was made about 2 cm below the right lower eyelid at an area of maximal fluctuance. The right face was copiously irrigated with normal saline, and drains were secured (Figure 3). Cautery was avoided due to the potential for OR fire given the caustic nature of the injected substance. He returned to the Surgical Intensive Care Unit (SICU) on a broadened antibiotic regimen with steroids continued.

Given ongoing necrosis, the patient was taken back to the operating room for serial irrigation and debridement over the next two weeks, for a total of seven operations. This required a multidisciplinary approach, including irrigation and debridement of the retrobulbar space with ophthalmology and the neck with ENT. Intra-operative cultures grew methicillin-sensitive *Staphylococcus aureus* (MSSA), methicillin-sensitive *Staphylococcus epidermidis* (MSSE), and *Cutibacterium acnes*, for which the infectious disease team initially placed him on a nine-day course of Zosyn/Linezolid, followed by an 11-day course of Unasyn/Linezolid. No antibiotics were recommended on discharge. Despite the patient’s chemical cellulitis with mild right-sided diplopia, his vision and ocular function remained grossly intact. Given the progression of neck swelling prior to his initial OR visit, along with the subsequent serial trips to the operating room, he underwent a prolonged period of intubation. He was ultimately extubated on hospital day 13 and was discharged home on hospital day 20. His course was otherwise complicated by a subsegmental pulmonary embolus, for which he completed a three-month course of Eliquis.

**Figure 4:**
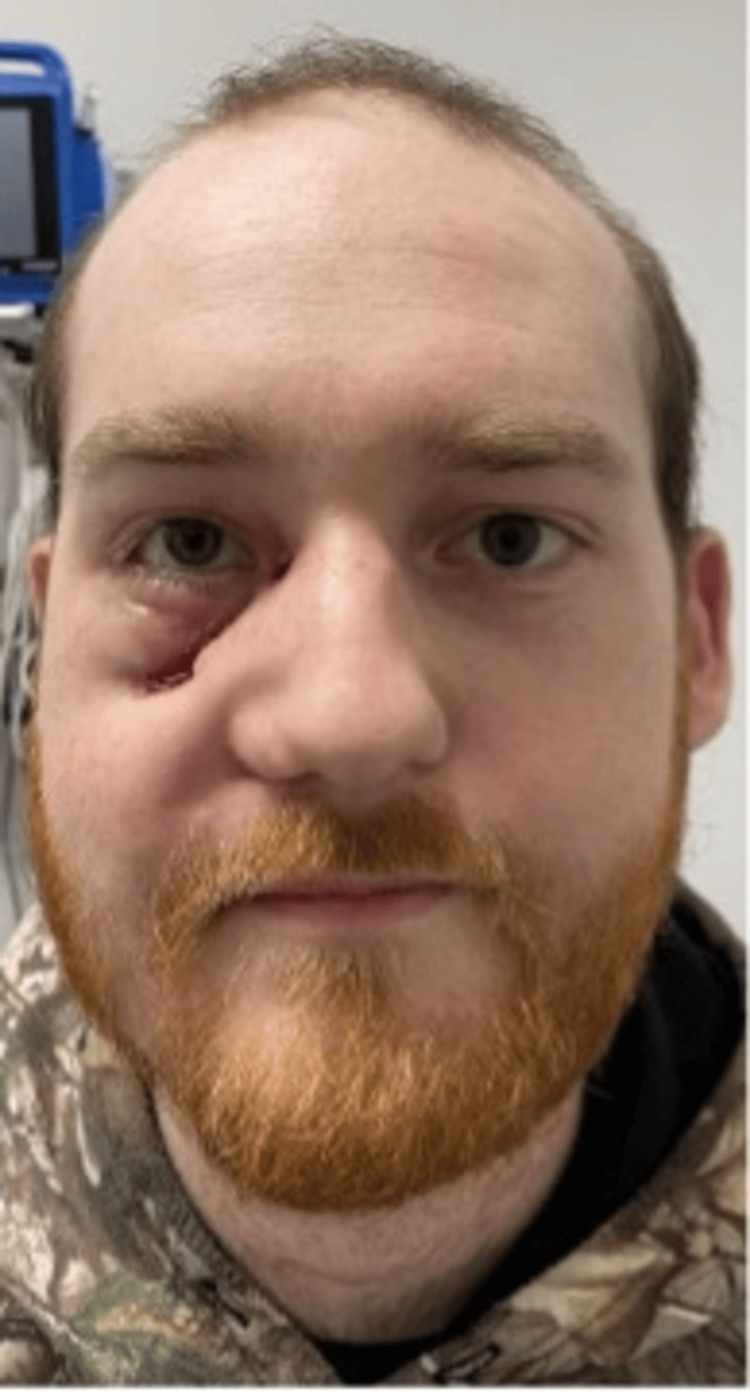
Patient at follow-up visit eight weeks after injury demonstrating R cheek soft tissue defect with inferior lid ectropion

**Figure 5:**
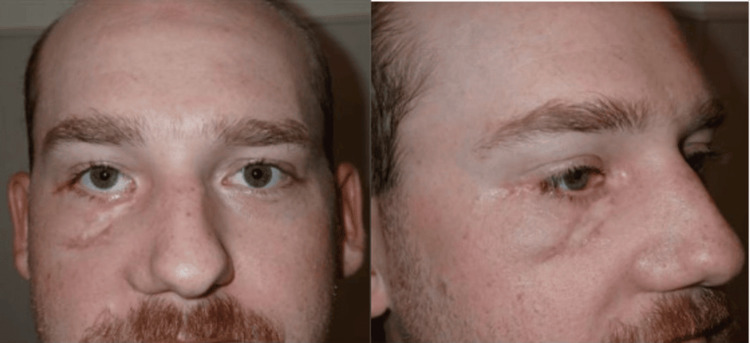
The patient at follow-up visit six months after injury following release of the right lower lid ectropion and full-thickness skin graft

He was followed closely in the outpatient setting by both plastic and oculoplastic surgery. At his initial follow-up visit two weeks post discharge, his exam was notable for a right 4 x 1 cm wound at the right lid-cheek junction, as well as a right lower eyelid ectropion with excess scleral show medially (Figure 4). Additionally, he had a right facial nerve palsy of the buccal and zygomatic branches. He was otherwise doing quite well. He was seen by ophthalmology for the ectropion, with a plan for delayed intervention given the need for three months of anticoagulation. Three months after discharge, he returned to the OR with both plastic and oculoplastic surgery for release and repair of his right lower eyelid ectropion and full-thickness skin graft from the right supraclavicular area. At his most recent outpatient visit, six months after discharge, both his ectropion and facial nerve function were improved. He reported he was doing well and no longer had pain (Figure 5).

## Discussion

Pressurized injection injuries to the face are highly unusual and thus scarcely reported in the medical literature. The treatment approach has largely been extrapolated from injection injuries to the hand. Hand injection injuries are also uncommon but have been well-described in the hand literature with a consensus regarding the treatment algorithm and strategies [2]. While the injury mechanism and many of the management principles are the same, there are several key differences and nuances regarding facial injection injuries that warrant discussion.

**Figure 6:**
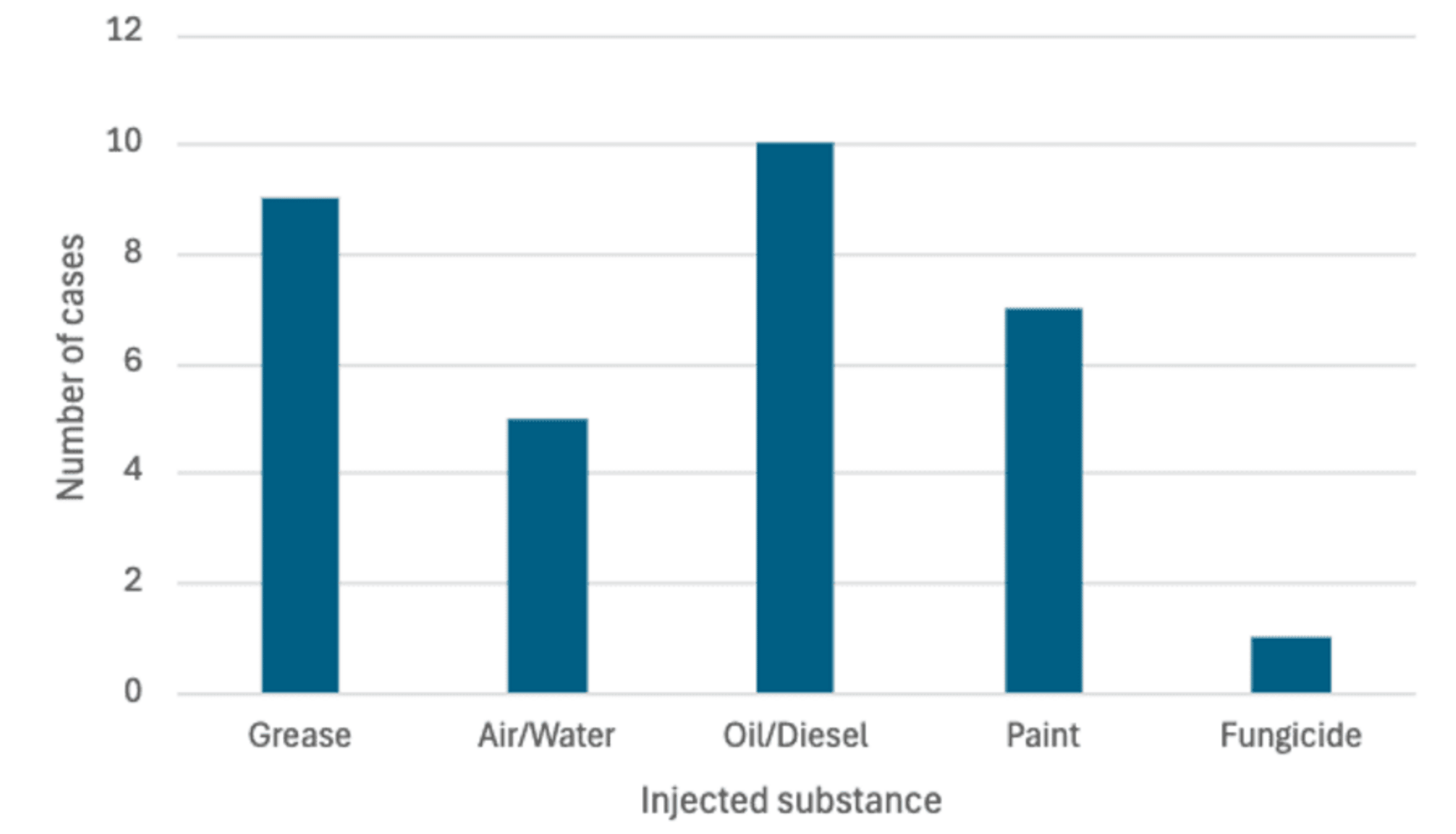
Facial injection injuries by substance This image was created by the corresponding author of this article.

When assessing facial injection injuries, it is critical to determine the injury mechanism, which includes the substance injected, the pressure measured in pounds per square inch (PSI), the volume of injected material, and the site of injection. More caustic substances, such as diesel or paint, should be treated the most aggressively [3]. In the hand literature, the likelihood of amputation correlates with the material injected, with turpentine and paint having the highest rates of amputation [4,5]. Grease gun injuries, which can also be severe, are associated with less chemical damage [6,7]. Based on our review of the literature, the most commonly injected substances were diesel/oil and grease (Figure 6). The following discussion reviews the pathophysiology and management of facial injection injuries for various substances and concludes with general management principles.

Grease gun injuries to the face have been reported as early as 1964 and are known to cause damage via both mechanical and chemical insults [1]. Grease is a highly viscous substance emulsified with mineral oil with low destructive properties, but under high pressure, it can diffuse along fascial planes, muscles, and neurovascular structures. The ensuing chemical irritation can lead to granulomatous inflammation, fibrosis, tissue necrosis, and sinus formation. For injection injuries around the orbit, the most common findings are periorbital or eyelid swelling, pain, and proptosis [8]. Treatment delay is common, particularly in cases without a visible entry wound or a small lesion that has already healed. There are reports of grease gun injuries presenting in a delayed fashion, but still necessitating operative debridement [9]. For grease gun injection injuries, most authors advocate for immediate surgical exploration and debridement [8]. Despite these recommendations, most cases of facial grease injections in the literature report operative debridement occurring at least two days post injury, though, in some cases, patients presented in a delayed fashion [6-8].

There are several reports of paint gun injuries to the face, which can be particularly challenging to treat [5,10,11]. As with all high-pressure injection injuries, the foreign material can be dispersed far from the entry point. Compared to grease, paint is less viscous and more toxic and destructive to local tissues and may also be more likely to contain bacteria [10,12]. These factors promote a more robust acute inflammatory response, promoting edema and potential compressive injuries. Ocular involvement can be devastating and must be carefully assessed via clinical exam and cross-sectional imaging. Given the toxicity of paint, early operative debridement and washout are even more critical than in grease injuries [10]. Debridement of these injuries should take place in the operating room to allow for a complete washout of as much foreign material as possible. Multiple case reports describe patients being debrided in the emergency department, only to decompensate shortly thereafter, requiring a trip to the operating room [4]. Failure to fully evacuate paint from the face and orbit can have long-term sequela, including granulomatous foreign body reactions that may manifest years later. Another consideration in paint injection injuries is the type of detergent used to aid in paint removal during washout. Historically, the mainstay has been normal saline or Ringer's lactate. Based on cadaveric studies in the hand literature, oil-based or latex-based paint injections should be irrigated with either povidone-iodine (PVP) prep or Johnson & Johnson baby shampoo. Saline irrigation alone may lead to incomplete washout, contributing to high reoperation rates [13].

High-pressure oil or diesel injection injuries to the face were the most frequently reported type of facial injection injuries in our literature review and carry significant morbidity. In 2022, Dawson et al. reported a similar case to ours involving eight trips to the operating room, with the patient ultimately requiring a sub-total orbital exenteration. Diesel fuel injectors can reach pressures up to 30,000 PSI, easily dissecting through tissue planes and triggering an inflammatory reaction that may lead to tissue necrosis and infection [3]. Hydrocarbons that are retained in tissues often lead to lipogranuloma formation, which will ultimately require excision [14]. Given the high velocity of fluid released from these hydraulic machines, the foreign material can easily traverse soft tissues and breach the orbital septum [3,14]. Pockets of fuel may be confused with orbital emphysema, as both appear hypodense on CT scan. As with all injection injuries, the initial presentation and small entry wound may lead the clinician to vastly underestimate the degree of underlying injury, as seen in this case.

Injections of air or water may be treated more conservatively [3,15]. However, given that water sprays can generate up to 55,000 PSI, these injuries can still penetrate through fascial planes and damage neurovascular structures [15]. The infiltration of air and water into tissue may result in significant subcutaneous emphysema seen on the CT scan and can lead to elevated compartment pressures. Additionally, the water used in high-pressure water jets may be obtained from rivers, reservoirs, lakes, or other environmental sources, which can be associated with a wide array of microorganisms. Microbacteria, notably *Vibrio vulnificus* seen in saltwater and *Aeromonas *spp. seen in freshwater, can lead to wound infections and potentially necrotizing fasciitis [16]. Broad-spectrum antibiotics should be started as prophylaxis against organisms found in nonsterile water and soil [15]. Fungal species may also be present in stagnant water, treated tap water, and in soil, and consideration should be given for antifungal coverage [16]. Three of the five total reported cases of air or water injection injuries to the face were treated conservatively, with one case requiring a single trip to the operating room for repair of conjunctival lacerations, and the other injury found to be superficial and closed primarily [15,17-19].

High-pressure injection injuries often present with minimal superficial skin changes, which can mask severe damage to the deeper structures. A thorough craniofacial exam should be performed at the initial encounter, including an assessment of the facial nerve, which can suffer mechanical or chemical damage. The initial exam must also include an ocular exam, with a low threshold for an Ophthalmology consult. Cross-sectional imaging with either CT or MRI should be performed to evaluate the extent of soft tissue involvement, with particular attention to the orbit, as well as the distribution of any foreign material [10,12]. For injuries involving the orbit, there is a risk of globe injury, retinal detachment, optic nerve injury, and intraconal hematoma [10].

**Figure 7:**
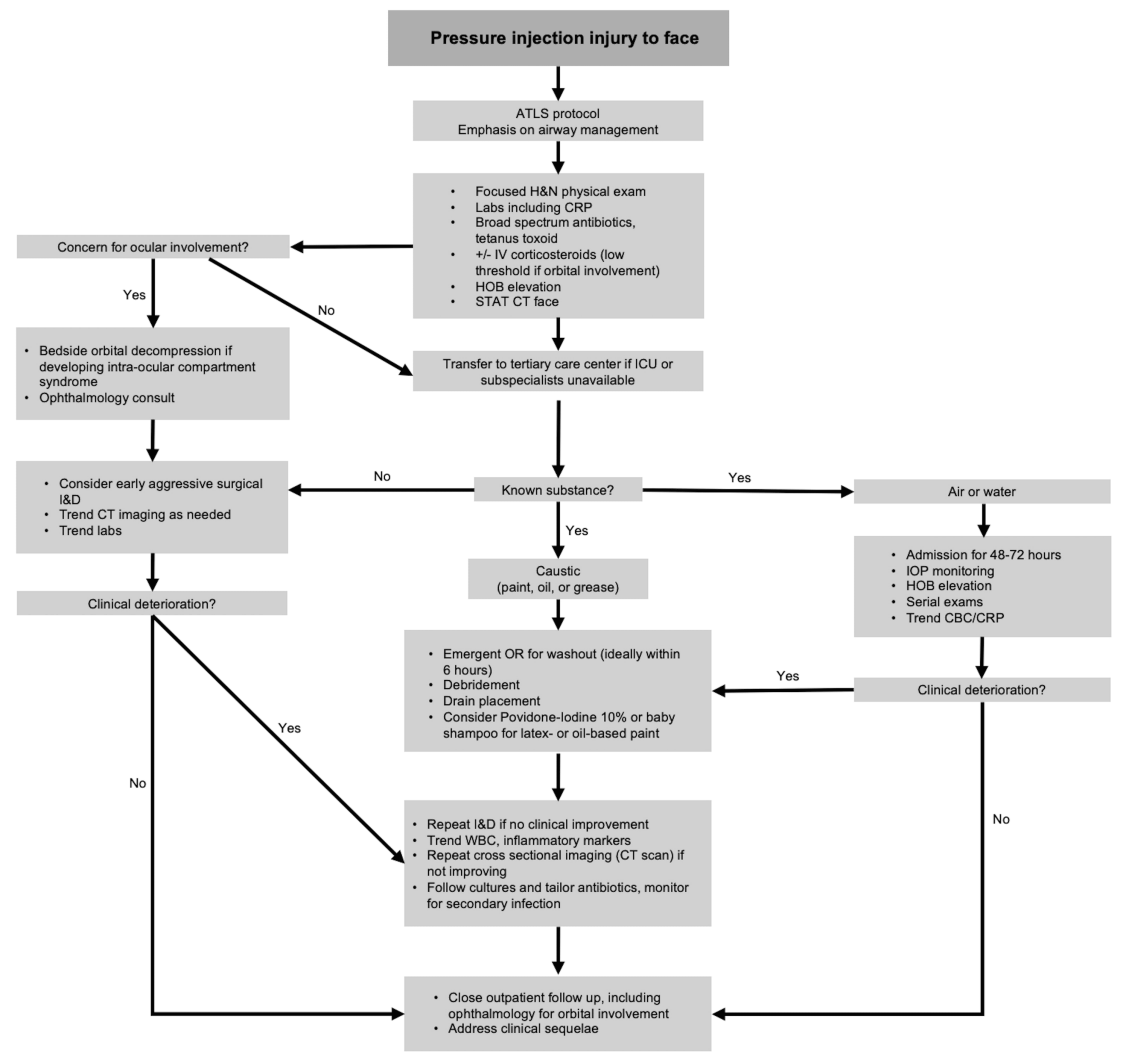
Proposed workup and treatment algorithm for facial injection injuries ATLS: Advanced Trauma Life Support. H&N: Head and Neck. CRP: C-reactive protein. IV: Intravenous. CT: Computed Tomography. ICU: Intensive Care Unit. IOP: Intraocular Pressure. HOB: Head of Bed. CBC: Complete Blood Count This image was created by the corresponding author of this article.

Once the mechanism of injury has been clearly defined and imaging obtained to evaluate the extent of injury, the key tenet of the treatment algorithm is early debridement and thorough irrigation in the operating room, ideally within six to eight hours (Figure 7). However, aggressive debridement of the delicate facial structures may not always be feasible, particularly the orbital structures [14]. Orbital injuries can be uniquely challenging given the wide array of signs and symptoms that may be present. If there is any concern for ocular involvement, the ophthalmology team should urgently evaluate, and orbital decompression, if indicated, should not be delayed [20]. Additionally, the post-injury inflammatory response within the orbit can be robust, with evolving exam findings over a short period of time [4]. Therefore, these patients must be monitored very closely. Occasionally, patients who have suffered injection injuries will present in a delayed fashion, with symptoms of pain, swelling, or erythema. Even in this setting, there should be a low threshold for operative debridement.

Following initial surgical washout and careful debridement of necrotic tissue, patients should be closely monitored in the ICU. Broad-spectrum antimicrobials should be continued and tailored based on specimens obtained from initial debridement. Inflammatory markers such as C-reactive protein should be trended daily. They may be a useful indicator of ongoing inflammation and possible retained foreign material and aid in the decision to return to the operating room [3]. Steroids are often given in facial injection injuries and should be strongly considered in cases involving the orbit and optic nerve, which may reduce edema and inflammation [7,12,14]. Consideration should also be given to repeat cross-sectional craniofacial imaging (CT scan) if there is any clinical deterioration with concern for undrained foreign material or abscess formation that would necessitate a washout.

## Conclusions

To optimize outcomes in high-pressure facial injection injuries, particularly those involving caustic substances, an aggressive surgical approach is needed, with attention to orbital involvement. Additionally, these patients should be treated in an ICU and monitored closely for the development of secondary infections, with a low threshold to return to the OR as needed. Secondary injury and long-term sequelae requiring reoperation are common, so patients should be followed closely in the outpatient setting.
